# Leadership behaviour in preventing and reducing workplace loneliness and social isolation in healthcare: a scoping review

**DOI:** 10.1108/LHS-08-2025-0131

**Published:** 2026-04-02

**Authors:** Outi Kanste, Minna Ylisirniö, Eeva Vuorivirta-Vuoti, Suvi Kuha

**Affiliations:** Research Unit of Health Sciences and Technology, University of Oulu, Oulu, Finland; The Finnish Centre for Evidence-Based Health Care: A Joanna Briggs Institute Centre of Excellence, Helsinki, Finland and Medical Research Center Oulu, Oulu, Finland; Research Unit of Health Sciences and Technology, University of Oulu, Oulu, Finland, and The Finnish Centre for Evidence-Based Health Care: A Joanna Briggs Institute Centre of Excellence, Helsinki, Finland

**Keywords:** Healthcare, Leadership, Loneliness, Scoping review, Social isolation

## Abstract

**Purpose:**

This study aims to identify existing literature and the current evidence of leadership behaviour in preventing and reducing workplace loneliness and social isolation in healthcare.

**Design/methodology/approach:**

A scoping review was conducted following the Joanna Briggs Institute methodology. In January 2025, 6,600 studies were screened after being retrieved from seven databases (CINAHL, Ovid Medline, ProQuest, Scopus, Web of Science, Finnish database Medic and MedNar). Five independent reviewers screened studies according to the inclusion criteria. Published and unpublished studies in English, German, Finnish and Swedish with no time limit were included. Data were synthesised using thematic analysis.

**Findings:**

A total of 16 studies published between 2000 and 2025 were included, and four themes in leadership behaviour in preventing and reducing workplace loneliness were identified: seeing the person as a human being, strengthening the sense of belonging, fostering interpersonal relations and enhancing sustainable working conditions.

**Practical implications:**

The results can inform the development of human-centred leadership, emphasising the importance of each professional’s contribution and the well-being of work communities to enhance professional retention. Leaders must identify their responsibilities and significant roles, as well as the training and systematic support needed to manage demanding leadership tasks.

**Originality/value:**

Healthcare leaders can significantly reduce and prevent workplace loneliness and social isolation. By highlighting individuals’ meaningfulness and contributions, supporting relationships within work communities, and promoting sustainable working conditions, leaders can enhance employees’ sense of value and belonging within the organisation.

## Introduction

Population ageing, increased demand for healthcare services, and shortages of skilled professionals have challenged healthcare systems worldwide (WHO, 2023). At the same time, rapid organisational changes and evolving work practices – such as digitalisation and reduced face-to-face interaction –have been linked to increased risks of loneliness and social isolation in the workplace (Kolcz *et al.*, 2023; Meese *et al.*, 2024; O'Hare *et al.*, 2024). Thus, it has become increasingly important for healthcare organisations to cultivate leadership behaviours that prevent and reduce workplace loneliness and social isolation, thereby supporting workforce well-being and promoting healthcare system sustainability.

Leadership can be conceptualised as a process through which leaders engage in behaviours that influence others towards attaining common goals (Northouse, 2021). Kouzes and Posner (2023) identify five core leadership behaviours: modelling the way, inspiring a shared vision, challenging the process, enabling others to act, and encouraging the heart. These behaviours can be examined through leadership styles, which offer a broader framework for understanding how leaders characteristically enact such behaviours in practice (Northouse, 2021). Thus, leadership plays a significant role in shaping the employees’ behaviours in work communities. According to prior studies, servant leadership enhances compassion and reduces workplace bullying (Ahmad *et al.*, 2023), and responsible leadership supports work engagement through knowledge sharing (Ali *et al.*, 2025). Leadership aligning individuals encompasses behaviours related to communication, commitment and team-building – behaviours that foster social connections within the workplace and play a crucial role in reducing loneliness and social isolation (Meese *et al.*, 2024). Within the framework of this scoping review, leadership behaviour is grounded in the ethical responsibility of leaders to recognise and address the needs and concerns of their followers (Northouse, 2021), particularly given the strong evidence of the adverse impacts of loneliness and isolation on organisational performance and employee well-being (Kolcz *et al.*, 2023; Meese *et al.*, 2024; Stubbs and Achat, 2022).

Loneliness is a form of emotional distress characterised by a lack of meaningful relationships, regardless of the size of one’s social network. In contrast, social isolation is an objective condition defined by a lack of social connections, which can be quantitatively assessed. Loneliness and social isolation are conceptually intertwined and often studied together (Kolcz *et al.*, 2023). Workplace loneliness refers to a negative psychological experience arising from interpersonal relationships at work that do not meet an individual’s expectations, both in terms of quality and quantity (Wright *et al.*, 2006). Similarly, Deniz (2019) defines workplace loneliness as a sense of social deprivation and disconnection characterised by difficulties forming social relationships, a lack of understanding from colleagues, and an overall sense of social distance within the work environment.

Research evidence demonstrates that both workplace loneliness (Kolcz *et al.*, 2023; Meese *et al.*, 2024; Stubbs and Achat, 2022; Wax *et al.*, 2022) and social isolation in the workplace (Meese *et al.*, 2024; O'Hare *et al.*, 2024) can have significant negative consequences for employees and organisations. Loneliness in the workplace markedly impacts the job performance of hospital employees (Deniz, 2019; Stubbs and Achat, 2022; Wax *et al.*, 2022). Furthermore, workplace loneliness and social isolation are associated with detrimental effects on affective organisational commitment, perceptions of colleagues and leaders (Wax *et al.*, 2022), and organisational citizenship behaviours (Wax *et al.*, 2022). These issues profoundly influence working behaviour, attitudes, productivity (Stubbs and Achat, 2022) and the overall well-being of healthcare workers (O'Hare *et al.*, 2024). Many of these consequences are linked to mental health disorders (Stubbs and Achat, 2022) as they lead to decreased job satisfaction (Meese *et al.*, 2024) and resilience (Meese *et al.*, 2024; Stubbs and Achat, 2022). In addition, they heighten the risk of burnout (Meese *et al.*, 2024), elevated distress (Meese *et al.*, 2024; Stubbs and Achat, 2022), increased turnover rates (Stubbs and Achat, 2022) and a variety of health conditions, including depression (Stubbs and Achat, 2022; Kolcz *et al.*, 2023), anxiety, physical inactivity (Stubbs and Achat, 2022), exhaustion and burnout (Kolcz *et al.*, 2023) among healthcare workers.

The influence of professional identification and social support on alleviating workplace loneliness is well-documented (O'Hare *et al.*, 2024). However, research on the role of leadership behaviour in preventing workplace loneliness and social isolation remains limited, and there is a significant lack of interventions to address these issues. A preliminary search of PROSPERO, Ovid Medline, the Cochrane Database of Systematic Reviews and JBI Evidence Synthesis in May 2024 identified no current scoping or systematic reviews on this topic.

Consequently, there is a pressing need for a systematic examination of leadership behaviour that can prevent and reduce workplace loneliness and social isolation in healthcare settings, as existing evidence remains fragmented and conceptually inconsistent. Thus, this review aimed to identify the existing literature and current evidence on leadership behaviour in preventing and reducing workplace loneliness and social isolation in healthcare. By synthesising the available evidence, this review clarifies what is currently known and provides direction for future research and intervention development. The review question addressed was:

*RQ1*. What leadership behaviour can prevent and reduce workplace loneliness and social isolation in healthcare?

## Research methods

### Study design

The scoping review was chosen as the methodology because it is well-suited to identifying evidence on the research topic, mapping the current research literature and demonstrating gaps in research knowledge. The review protocol has been registered in the Open Science Frame database (*blinded for peer review*). The review followed the Joanna Briggs Institute’s (JBI) methodology for scoping reviews (Peters *et al.*, 2020). The Preferred Reporting Items for Systematic Reviews and Meta-analyses extension for Scoping Reviews (PRISMA-ScR) checklist (Tricco *et al.*, 2018) was used to report the results (Supplementary Table 1).

### Data sources and search strategies

A three-step search strategy was used to locate published and unpublished studies (Peters *et al.*, 2020). An initial limited search of Ovid Medline and Scopus was conducted, followed by an analysis of the text words in the title and abstract, as well as the index terms used to describe the study. On this basis, a complete search strategy was developed and tailored to each information source. The information specialist assisted in developing the search strategy. Then, a second search was conducted across seven databases, including CINAHL, Ovid Medline, ProQuest, Scopus, Web of Science and the Finnish database Medic. The search for unpublished studies and grey literature included MedNar. The entire search was completed in January 2025. Studies published in English, German, Finnish, and Swedish were included because the research team had the necessary language skills. No time limit was used in the data searches. (Supplementary Table 2).

### Study selection

Inclusion and exclusion criteria were defined based on the PCC (participants, concept and context) framework (Peters *et al.*, 2020), as detailed in Table 1. A search of seven databases resulted in 6,600 records. After the search, all identified citations were collated in Covidence Systematic Review Software (2023), and duplicates were removed. After removing duplicates, 4,553 records were screened by title and abstract, of which 4,495 were subsequently excluded. The titles and abstracts were independently screened by five reviewers (*blinded for peer review*) to assess their alignment with the inclusion criteria.

**Table 1.** Inclusion and exclusion criteria used in the scoping review

PCC	inclusion criteria	Exclusion criteria
Participants (P)	All professionals (e.g. nurses, physicians, midwives, therapists) and leaders and managers*	Students and patients
Concept (C)	Any leadership behaviours, regardless of their nature (e.g. positive, negative, dark), for preventing and reducing workplace loneliness and social isolation	Does not apply leadership behaviours concerning workplace loneliness and social isolation
Context (C)	All healthcare settings and organisations in all geographic locations and cultural groups	Other than healthcare settings
Type of studies	Qualitative, quantitative and mixed methods study designs	Other types of publications, such as systematic and other types of reviews, opinion papers, editorials and theoretical papers
	Grey literature: conference proceedings and doctoral theses	

Note(s):

*At least 50% of the participants must be healthcare workers. If the study included multiple groups, only results from studies that compared healthcare professionals or leaders with managers from other groups were used

**Source(s):** Authors’ own work

**Figure 1.**
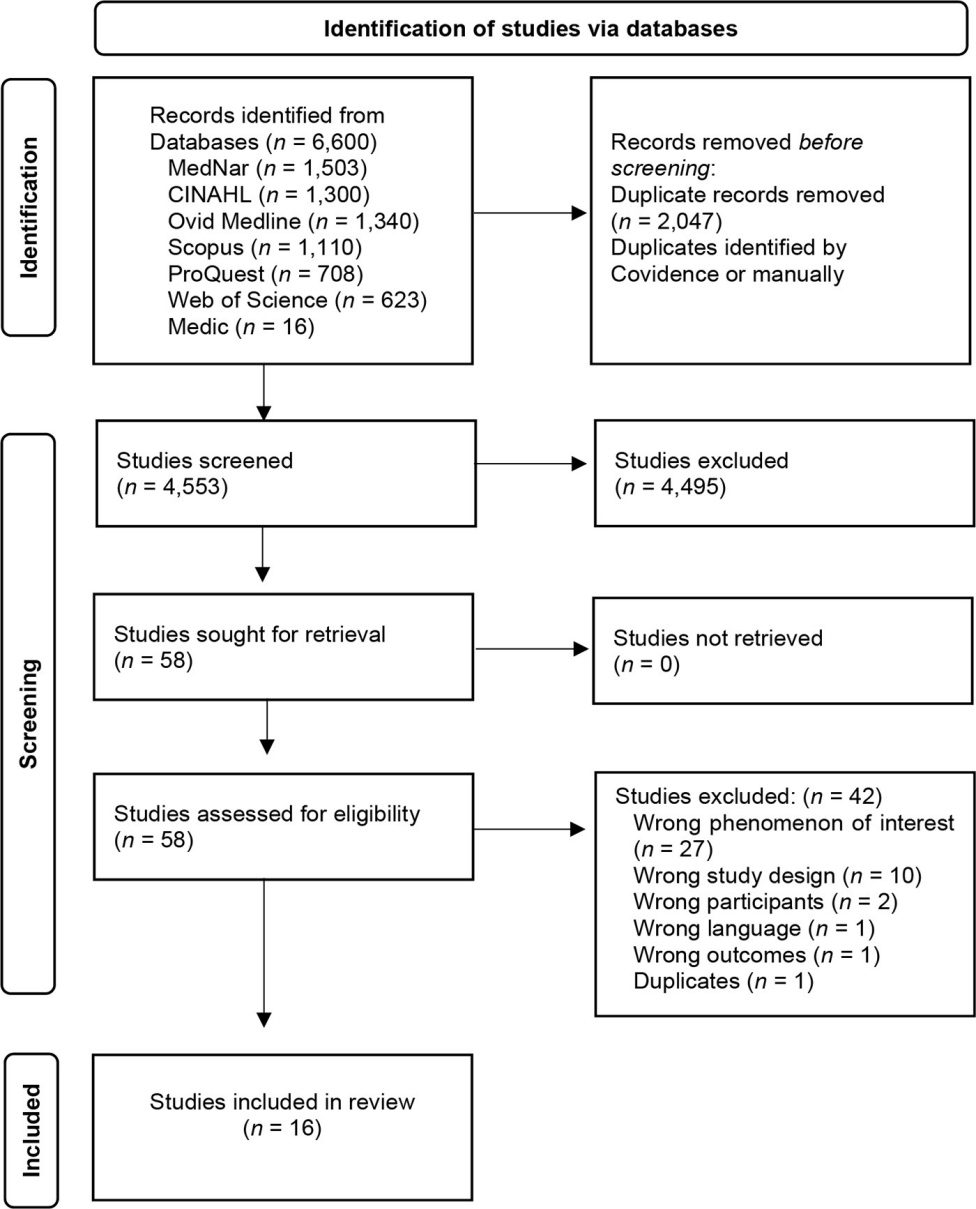
PRISMA flowchart of the search results, study selection and inclusion process **Source:** Authors’ own work

Studies that met the inclusion criteria (*n* = 58) were retrieved, and 42 were excluded as they did not meet the eligibility criteria (Supplementary Table 3). Any disagreements with the reviewer were resolved through discussion or with the assistance of a third reviewer. Finally, the reference lists of the 16 full-text studies that met the inclusion criteria were reviewed, but no additional studies were identified. (Figure 1).

### Data extraction and synthesis

A total of 16 studies were included in this review, and relevant data and descriptive information were extracted using a data extraction tool developed by the researchers following the guidelines for scoping reviews by JBI (Peters *et al.*, 2020). The data extraction tool included specific details about the authors, publication year, country of origin, study purpose/aim, research design, data collection and analysis, participants (sample size), study context and key findings (Supplementary Table 4). Four reviewers (*blinded for peer review*) independently extracted data, and the recordings were subsequently discussed within the research group. Any disagreements between the reviewer and the authors were resolved through discussion (Peters *et al.*, 2020).

Thematic analysis was used to synthesise the main findings (Thomas and Harden, 2008). The data synthesis was conducted by four researchers (blinded for peer review). Firstly, free-line-by-line coding of findings from primary studies was conducted to facilitate the translation of concepts across studies. Next, these free codes were organised into related areas to construct descriptive themes. Finally, descriptive themes were grouped into subthemes and used to develop analytical themes. The findings were reported using explanatory text and tabular format (Peters *et al.*, 2020).

## Findings

### Overview of the reviewed studies

The review encompasses 16 studies published between 2000 and 2025, conducted across 11 countries. These countries include Australia (*n* = 2), Korea (*n* = 1), Finland (*n* = 2), Norway (*n* = 1), France (*n* = 1), Romania (*n* = 1), Sweden (*n* = 1), Saudi-Arabia (*n* = 1), Turkey (*n* = 1), the UK (*n* = 2) and the USA (*n* = 3). Nine studies used qualitative research methods, with most data collected through individual and focus group interviews. Six studies used quantitative methods, most of which were cross-sectional surveys. One study reported the results of a mixed-methods study.

The studies covered participants from different healthcare settings. Nurses (*n* = 8), clinical and healthcare staff (*n* = 4) and physicians (*n* = 3) were the most frequently studied participant groups. The number of participants ranged from 4 to 1,829, for a total of 3,997.

The studies concerning leadership behaviour in preventing and reducing workplace loneliness and social isolation in healthcare covered the following four main themes: 1) seeing the person as a human being, 2) strengthening the sense of belonging, 3) fostering interpersonal relations, and 4) enhancing sustainable working conditions (Table 2).

**Table 2.** An overview of descriptive themes, subthemes and analytical themes

Descriptive themes (*n* = 24)	Subthemes (*n* = 9)	Analytical themes (*n* = 4)
Enabling equality in leadership Implementing diversity leadership	Valuing ethical leadership	Seeing the person as a human being
Emphasising the elements of a leaderr- member social exchange Endorsing mutual recognition between leaders and professionals Enhancing the conscious presence of leaders	Strengthening the relationship between the leader and the professional	
Onboarding to work Guidance for work Attachment to organisation	Promoting socialisation in the organisation	Strengthening the sense of belonging
Making participation possible Being an integral part of the work community Feeling like an outsider	Enabling the sense of involvement	
Fostering interprofessional commonality Enhancing multidisciplinary collaboration Noticing the isolated nature of work	Improving communal ways of working	Fostering interpersonal relations
Ensuring communication Enabling daily interaction between work community Creating an open working culture	Enabling high-quality interaction in the work community	
Peer support Support from the leader	Creating supportive networks for professionals	Enhancing sustainable working conditions
Observing professionals’ well-being Appropriately allocating work tasks Assessing the impact of organisational structure on work	Ensuring quality working conditions	
Meaningfulness of work Recognition of achievements and merits	Valuing and appreciating work	
		

**Source(s):** Authors’ own work

### Seeing the person as a human being

The first main theme, seeing the person as a human being, included two subthemes: valuing ethical leadership and strengthening the relationship between leader and professional.

*Valuing ethical leadership* includes enabling equality in leadership (Crawford *et al.*, 2005) and implementing diversity leadership within the work community (Dreachslin *et al.*, 2000).

Enabling equality in leadership involves recognising the equal significance of all professionals, regardless of their employment contract, job title, or personal characteristics, thereby reinforcing a sense of inclusivity and recognition as equal members of the work community (Crawford *et al.*, 2005). Furthermore, implementing diversity leadership by providing opportunities for intercultural interaction and proactive leadership in addressing selective perception and stereotyping is critical to enhancing ethical leadership and preventing social isolation (Dreachslin *et al.*, 2000).

*Strengthening the relationship between leaders and professionals* includes emphasising the elements of Leader-Member Social Exchange (Arslan *et al.*, 2020), endorsing mutual recognition between leaders and professionals (Arslan *et al.*, 2020; Flood *et al.*, 2022; Huyghebaert *et al.*, 2018; Wood *et al.*, 2023) and enhancing the conscious presence of leaders (Flood *et al.*, 2022; Wood *et al.*, 2023; Førsund and Schumacher, 2023). According to Arslan *et al.* (2020), the elements of Leader-Member Social Exchange (LMSX), which conceptualise this relationship through dimensions of mutual trust, diffuse obligation, long-term orientation and social-emotional exchanges, can significantly mitigate feelings of isolation in the workplace.

Furthermore, in preventing loneliness and social isolation, leaders must engage in practices that endorse mutual recognition between themselves and professionals, such as addressing appreciation work (Flood *et al.*, 2022; Wood *et al.*, 2023), deepening understanding of the content of professionals’ work (Wood *et al.*, 2023), enhancing nurses’ affective commitment (Huyghebaert *et al.*, 2018) and promoting trust in leaders (Arslan *et al.*, 2020). Feelings of appreciation are particularly significant in reinforcing individuals’ sense of being seen as whole people rather than mere resources (Flood *et al.*, 2022; Wood *et al.*, 2023). The COVID-19 pandemic highlighted the detrimental effects of insufficient recognition of leaders, with acute care professionals feeling dehumanised and treated as expendable resources. Many also perceived a lack of understanding from their leaders regarding the content of their work, as leaders were distanced from daily clinical practice (Wood *et al.*, 2023; Flood *et al.*, 2022). In this context, Huyghebaert *et al.* (2018) found that feelings of isolation were negatively associated with nurses’ affective commitment to their leaders. Arslan *et al.* (2020) demonstrated that trust in leadership was a statistically significant predictor of workplace loneliness. Specifically, nurses with lower trust in their leaders reported higher levels of loneliness, further emphasising the importance of leader engagement in fostering trust and mutual recognition.

Strengthening the relationships with professionals by enhancing leaders’ conscious presence within the work community (Flood *et al.*, 2022; Wood *et al.*, 2023; Førsund and Schumacher, 2023) is important as a lack of presence and accessibility of leaders has been shown to increase feelings of loneliness and social isolation especially during the COVID-19 pandemic (Førsund and Schumacher, 2023; Wood *et al.*, 2023). Throughout the COVID-19 pandemic, acute care professionals expressed concerns that leaders were increasingly detached from everyday practice (Wood *et al.*, 2023). Similarly, home care professionals reported being left to navigate organisational issues independently due to their leaders’ remote working arrangements (Førsund and Schumacher, 2023). Throughout the pandemic, leaders acknowledged their responsibility for being present for professionals (Flood *et al.*, 2022). Minimising the distance between themselves and professionals (Wood *et al.*, 2023), fostering accessibility through open-door practices (Flood *et al.*, 2022), and actively being present and taking responsibility for organisational issues (Førsund and Schumacher, 2023) can enhance the relationship between the leaders and professionals.

### Strengthening the sense of belonging

Strengthening the sense of belonging included two subthemes: promoting socialisation in the organisation and enabling the sense of involvement.

*Promoting socialisation in the organisation* includes onboarding (Aira *et al.*, 2010), guidance at work (Aira *et al.*, 2010) and attachment to the organisation (Huyghebaert *et al.*, 2018; Stoica *et al.*, 2014). Recognising the importance of effective onboarding for new employees is essential for preventing and reducing workplace loneliness and social isolation. For example, physicians in primary health care centres have reported feeling isolated during the early days of their careers due to insufficient orientation. In addition, guidance to work, such as mentoring and tutoring, can significantly impact experiences of loneliness. Mentoring has been identified as a key factor in reducing loneliness among General Practitioners (GPs) in the workplace. However, it is noteworthy that GPs have had minimal exposure to formal mentoring. Alternatively, tutoring can be used to reduce loneliness and social isolation. For example, junior doctors and senior GPs could pair up as one-on-one tutors to reduce feelings of loneliness (Aira *et al.*, 2010). Supporting professionals’ attachment to the organisation can reduce workplace loneliness, as employees with high loneliness tend to have low organisational attachment. Attachment can be strengthened with the support of leaders and senior employees (Stoica *et al.*, 2014). In addition, Huyghebaert *et al.* (2018) found that nurses who experience a stronger emotional bond with their organisation also identify more with their supervisor. However, this emotional bond is impaired when nurses feel socially isolated from their co-workers.

*Enabling the sense of involvement* consisted of making participation possible (Aira *et al.*, 2010), being an integral part of the work community (Crawford *et al.*, 2005) and feeling like an outsider (Lindgren *et al.*, 2001). The leader can enable participation in the work community, which can be important in reducing workplace loneliness and social isolation. Studies have found that physicians are expected to contribute to their workplace and hope to be kept informed of what is happening in the workplace (Aira *et al.*, 2010). It has also been found that feelings of social isolation increase if professionals do not feel an integral part of the work community or if professional peers are absent from the work community. This has been found, for example, among dental hygienists, who were less likely than other professional groups to feel an integral part of a particular work community because they often worked in multiple locations or as part-time employees (Crawford *et al.*, 2005). Similar experiences have been found among physiotherapists who felt like outsiders from the primary healthcare organisation (Lindgren *et al.*, 2001).

### Fostering interpersonal relations

Fostering interpersonal relations included two subthemes: improving communal ways of working and enabling high-quality interaction in the work community.

*Improving communal ways of working* consisted of fostering interprofessional commonality (Aira *et al.*, 2010; Crawford *et al.*, 2005), enhancing multidisciplinary collaboration (Aira *et al.*, 2010; Mäntyselkä *et al.*, 2010; Cho and Kim, 2024) and noticing the isolated nature of work (Digby *et al.*, 2021; Førsund and Schumacher, 2023; Lindgren *et al.*, 2001; Yeh *et al.*, 2019; Wood *et al.*, 2023; Zomerdijk *et al.*, 2023). The leader needs to foster interprofessional commonality in the workplace, which can contribute to experiences of loneliness (Aira *et al.*, 2010; Crawford *et al.*, 2005). Studies have identified experiences of social isolation (Aira *et al.*, 2010) and unfair treatment (Crawford *et al.*, 2005) in interprofessional relationships in the workplace. Physicians have perceived themselves as socially isolated employees who are walked over by nursing staff who do not consider their personal needs and dictate their working pace. GPs also do not see the health system as a shared community and feel that hospital doctors only sometimes understand the working conditions in health centres (Aira *et al.*, 2010). Similarly, dental hygienists have experienced unfair treatment from other staff because other staff need to understand their responsibilities, education, and salary (Crawford *et al.*, 2005), highlighting the need for leaders to focus on fostering interprofessional commonality in the workplace.

It is also essential for the leader to enhance multidisciplinary cooperation, as insufficient multidisciplinary collaboration can lead to feelings of loneliness and social isolation (Aira *et al.*, 2010; Mäntyselkä *et al.*, 2010; Cho and Kim, 2024). The isolation experienced by physicians has been associated with perceived poor collaboration with nurses (Aira *et al.*, 2010; Mäntyselkä *et al.*, 2010), the nursing team, closest colleagues (Aira *et al.*, 2010) and the managerial team (Mäntyselkä *et al.*, 2010). Some physicians have experienced a lack of cooperation with nursing staff, which has caused difficulties in work, such as getting assistance in operations (Aira *et al.*, 2010). Furthermore, nurses in integrated nursing care services often feel lonely, as they perceive a lack of collaboration with professionals from other disciplines. They also feel that addressing patients’ needs has been disproportionately placed on them (Cho and Kim, 2024).

Managers must notice the isolated nature of work and consider how to increase the commonality between employees working independently, as the isolated nature of the work is linked to loneliness at work (Digby *et al.*, 2021; Førsund and Schumacher, 2023; Lindgren *et al.*, 2001; Yeh *et al.*, 2019; Zomerdijk *et al.*, 2023). Working in a physically separate building or floor from where the actual work was done has caused loneliness among physiotherapists and left them alone with the patient’s problem, without anyone to whom they can seek advice (Lindgren *et al.*, 2001). Similarly, the isolating nature of home care work (Førsund and Schumacher, 2023; Yeh *et al.*, 2019), physical distancing requirements (Zomerdijk *et al.*, 2023) like working in isolation rooms (Digby *et al.*, 2021) or silos (Zomerdijk *et al.*, 2023) and using personal protective equipment (Wood *et al.*, 2023) during the COVID-19 pandemic has been perceived as isolating and causing loneliness.

*Enabling high-quality interaction in the work community* consisted of ensuring communication (Aira *et al.*, 2010; Arslan *et al.*, 2020; Mäntyselkä *et al.*, 2010; Wood *et al.*, 2023), enabling daily interaction between the work community (Aira *et al.*, 2010; Crawford *et al.*, 2005; Mäntyselkä *et al.*, 2010; Wood *et al.*, 2023) and creating an open working culture (Aira *et al.*, 2010). Is it essential to ensure communication (Aira *et al.*, 2010; Arslan *et al.*, 2020; Mäntyselkä *et al.*, 2010; Wood *et al.*, 2023) by facilitating personal conversations with professionals (Aira *et al.*, 2010) and assuring that communication occurs adequately (Wood *et al.*, 2023) and frequently (Arslan *et al.*, 2020). In addition, cooperation between leaders and professionals should be ensured (Mäntyselkä *et al.*, 2010). Research indicates that GPs appreciate personal conversations with leaders, even though they often have not been in contact, met in person or even been able to name the members of their organisation’s management team (Aira *et al.*, 2010). Increased communication frequency between leaders and nurses is associated with reduced feelings of workplace loneliness (Arslan *et al.*, 2020). Furthermore, Wood *et al.* (2023) observed that adequate interaction from leaders can enhance professionals’ feelings of being recognised as whole individuals, although leaders’ interactions are often perceived as insufficient.

Daily interaction within the work community is essential, as a lack of interaction can be linked to feelings of loneliness and social isolation (Aira *et al.*, 2010; Mäntyselkä *et al.*, 2010; Wood *et al.*, 2023). Therefore, leaders need to facilitate formal and informal daily encounters between professionals during the working day (Aira *et al.*, 2010; Mäntyselkä *et al.*, 2010; Wood *et al.*, 2023). Poor collaboration with colleagues or other actors (Mäntyselkä *et al.*, 2010) and infrequent or absent opportunities to consult or meet colleagues during the working day are associated with GPs’ perceived social isolation at work (Aira *et al.*, 2010; Mäntyselkä *et al.*, 2010). Younger GPs often appreciate the opportunity to consult a more experienced colleague (Aira *et al.*, 2010). The lack of breaks has been identified as a crucial opportunity to connect with the work community and engage in confidential discussions with peers, which is essential when considering workplace loneliness (Crawford *et al.*, 2005; Aira *et al.*, 2010). Effective communication between peers promotes overcoming misunderstandings, which can lead to social isolation (Crawford *et al.*, 2005). Nurses have observed that even a brief opportunity to interact with colleagues during the workday can strengthen camaraderie and social support (Wood *et al.*, 2023). Supporting informal professional meetings can also be beneficial, as very concise GP communities that meet regularly and spend their free time together have been found to reduce loneliness (Aira *et al.*, 2010) and contact with colleagues outside the workplace has been found to strengthen internal relationships (Wood *et al.*, 2023).

Creating an open work culture provides formal opportunities for professionals to discuss problems and receive support from other team members, thereby reducing feelings of loneliness. It is essential to note that young GPs may hesitate to interrupt busy senior colleagues when they require consultation. On the other hand, senior GPs often worry that younger GPs might feel anxious about revealing their lack of knowledge (Aira *et al.*, 2010). This underscores the importance of leaders fostering an open feedback culture, as a lack of workplace feedback can lead to feelings of loneliness.

### Enhancing sustainable working conditions

Enhancing sustainable working conditions included three subthemes: creating supportive networks for professionals, ensuring quality working conditions and valuing and appreciating work.

*Creating supportive networks for professionals* consisted of peer support (Aira *et al.*, 2010; Lindgren *et al.*, 2001; Yeh *et al.*, 2019) and support from the leader (Digby *et al.*, 2021; Førsund and Schumacher, 2023; Stoica *et al.*, 2014; Yeh *et al.*, 2019; Wood *et al.*, 2023). Some studies have associated peer support with workplace loneliness (Aira *et al.*, 2010; Crawford *et al.*, 2005; Lindgren *et al.*, 2001; Yeh *et al.*, 2019). Forming supportive bonds with other workers may help alleviate loneliness (Yeh *et al.*, 2019) when cooperation is effective (Lindgren *et al.*, 2001). By focusing on peer support, social isolation in the workplace can be reduced (Aira *et al.*, 2010).

Support from the leader is essential to reduce workplace loneliness among professionals (Stoica *et al.*, 2014; Yeh *et al.*, 2019; Førsund and Schumacher, 2023). Also, the presence of leaders reduces social isolation when the work is generally isolated (Yeh *et al.*, 2019; Førsund and Schumacher, 2023). Leaders are essential in directing correct processes, listening to staff, and addressing issues, especially during the COVID-19 pandemic, when clinical staff experienced social and emotional isolation and the challenges of social distancing (Digby *et al.*, 2021). Leaders can also prevent workplace loneliness by enabling support from organisation psychologists (Stoica *et al.*, 2014) or by organising meetings with the chaplain (Wood *et al.*, 2023).

*Ensuring quality working conditions* involved observing professionals’ well-being (Huyghebaert *et al.*, 2018; Wood *et al.*, 2023; Yousef *et al.*, 2024), appropriately allocating work tasks (Aira *et al.*, 2010) and assessing the impact of organisational structure on work (Crawford *et al.*, 2005; Aira *et al.*, 2010; Mäntyselkä *et al.*, 2010; Førsund and Schumacher, 2023). Leaders can prevent social isolation in the workplace by observing professionals’ well-being. Studies have recognised that workplace isolation is negatively linked to nurses’ well-being but positively associated with nurses’ turnover intentions. In particular, the more nurses feel socially isolated from their colleagues, the lower their well-being and the more likely they are to intend to leave (Huyghebaert *et al.*, 2018). Also, the correlation between loneliness and burnout has been noted (Wood *et al.*, 2023; Yousef *et al.*, 2024).

Research has demonstrated that the appropriate allocation of work tasks by professionals can reduce feelings of loneliness in the workplace. It is essential to ensure effective distribution of work and maintain adequate work control among professionals. Specifically, the strategic distribution of tasks between physicians and nurses and the management of well-organised appointment schedules can help physicians retain control over their workload and reduce feelings of isolation (Aira *et al.*, 2010). Organisational structures have also been linked to workplace loneliness and social isolation (Crawford *et al.*, 2005; Mäntyselkä *et al.*, 2010; Aira *et al.*, 2010). Research indicates that physicians’ perceived isolation is associated with working in large health centres (Mäntyselkä *et al.*, 2010) or in remote satellite locations, where working alone can hinder their sense of community (Aira *et al.*, 2010). Similarly, Crawford *et al.* (2005) found that dental hygienists working across multiple units experience a lack of professional peers in the workplace, which can significantly contribute to feelings of loneliness.

Valuing and appreciating work included meaningfulness of work (Arslan *et al.*, 2020; Aira *et al.*, 2010) and recognition of achievements and merits (Aira *et al.*, 2010; Crawford *et al.*, 2005; Stoica *et al.*, 2014). The meaningfulness of work (Aira *et al.*, 2010; Arslan *et al.*, 2020) and the quality of leader-member exchanges have been negatively associated with workplace loneliness (Arslan *et al.*, 2020). This highlights the importance of providing support that enables employees to engage in work they find meaningful. Research indicates that a lack of recognition of achievements is associated with an increased risk of feelings of loneliness (Stoica *et al.*, 2014) and social isolation (Crawford *et al.*, 2005). Stoica *et al.* (2014) found a statistically significant positive correlation between a lack of recognition and feelings of loneliness among employees in medical units. Recognition fosters a sense of equality within the work community, reinforcing that individuals are valued equally and deserve the same benefits and acknowledgement as their peers (Crawford *et al.*, 2005). However, GPs working in primary health care have reported that hospital specialists sometimes undervalue their work, which can contribute to feelings of isolation and loneliness in the workplace (Aira *et al.*, 2010).

## Discussion

This is the first review to synthesise available evidence on leadership behaviour in preventing and reducing workplace loneliness and social isolation in healthcare. By integrating findings from 16 studies, four novel themes emerged: seeing the person as a human being, strengthening a sense of belonging, fostering interpersonal relations and enhancing sustainable working conditions.

Our findings indicate that professionals perceive their status and visibility within their work communities significantly influence their interpersonal relationships with the organisation or team. Focusing on the individual as a human being necessitates emphasising ethical issues in leadership and fostering a functional relationship between leaders and professionals. This can be achieved through conscious identification and the prevention of factors that challenge diversity, as well as by emphasising the importance of each professional to the work community and the organisation. The importance of equal recognition and the value of each professional contribution (O'Hare *et al.*, 2024) in fostering social connections within work communities has been found in previous studies to be particularly significant for groups at high risk of loneliness and social isolation (Deniz, 2019).

According to our review, the relationship between leaders and professionals also influences how professionals feel seen within their work communities. Consequently, leaders must have a comprehensive understanding of the content and circumstances of professionals’ work. To achieve this, leaders should cultivate strong relationships with professionals, demonstrate respect for them, and actively engage with the work community. Enhancing professionals’ commitment and gaining their trust should be a leadership priority in preventing loneliness and social isolation within work communities. The importance of leaders having a comprehensive understanding of issues relevant to professional work becomes particularly evident during periods of crisis. Such periods can intensify organisational pressures, including rapid changes in work practices, increasing digitalisation and reduced face-to-face interaction, and these have been linked to increased risks of loneliness and social isolation in the workplace (Kolcz *et al.*, 2023; Meese *et al.*, 2024; O'Hare *et al.*, 2024).

Our review findings indicate that the sense of belonging is contingent upon acquiring the requisite skills. Therefore, leaders should implement behaviours that facilitate the development of clinical skills while reinforcing individuals’ perceptions of community and organisational support. Such behaviour includes mentoring, which aims to build a personal and trusting relationship between mentor and mentee. In addition, tutoring, orientation, induction and organisational attachment promote organisational socialisation. A paucity of peers and organisational support has been identified as a potential cause of feelings of isolation, which, in turn, has been linked to negative perceptions of colleagues and leaders. (Wax *et al.*, 2022).

According to our review, a sense of belonging is also reinforced when individuals perceive a sense of involvement within the work community. Leaders can enhance professionals’ sense of belonging by cultivating an inclusive environment and proactively addressing factors that may contribute to feelings of isolation. This can be achieved by paying attention to how work is organised and fostering social connections within the work community. The social environment (Stubbs and Achat, 2022) and social distances in the work community (Deniz, 2019) have also previously been identified as significant contributors to feelings of loneliness, with a lack of social connections and meaningful relationships being particularly relevant (Kolcz *et al.*, 2023; Wright *et al.*, 2006).

Our review showed that the quality of relationships in work communities is pivotal in reducing and preventing loneliness and social isolation in healthcare. The findings highlighted communication, collaboration, and a sense of commonality as key areas for improvement, more clearly than previous findings. Prior studies show that understanding different leadership styles and their effects on knowledge sharing (Ali *et al.*, 2025) and compassion (Ahmad *et al.*, 2023) within work communities enables leaders to strengthen cohesion and communication among professionals. According to our review, leaders should prioritise enabling daily interaction within the work community. This is crucial, as previous studies have linked limited interaction and communication to social isolation and loneliness (Kolcz *et al.*, 2023; Meese *et al.*, 2024).

Our review found that enhancing sustainable working conditions, including supporting behaviours such as professional networks and organisational structures that promote good working conditions, is associated with workplace loneliness and social isolation. This includes the creation and reinforcement of behavioural norms, as well as the development of mechanisms for connection. The significance of this endeavour is underscored by findings that loneliness and social isolation have been identified as factors that can adversely affect job performance (Deniz, 2019; Stubbs and Achat, 2022; Wax *et al.*, 2022) and organisational commitment (Wax *et al.*, 2022). The findings of this review suggest that by closely monitoring the well-being of professionals, leaders can effectively predict the well-being of healthcare workers, as social isolation and workplace loneliness have been shown to have detrimental effects on these professionals’ well-being (O'Hare *et al.*, 2024; Stubbs and Achat, 2022). This, in turn, can lead to declines in productivity (Stubbs and Achat, 2022), job satisfaction (Meese *et al.*, 2024), and mental health risks (Kolcz *et al.*, 2023; Meese *et al.*, 2024; Stubbs and Achat, 2022) among these professionals.

### Limitations

This scoping review included some limitations that should be considered when interpreting the results. Although we conducted a thorough literature search under the guidance of an experienced information specialist, some relevant studies may have been missing. For example, this review only included studies in English, German, Finnish and Swedish. The review remains primarily descriptive, and there is a lack of evaluated interventions demonstrating the impact of leadership behaviour on reducing loneliness and social isolation, leaving uncertainty about what works in practice. In addition, the core concepts in this review are multifaceted and encompass various operational definitions that may influence the results. Because no critical appraisal was conducted, studies of differing quality are treated equally, creating a risk that low-quality or biased evidence may shape the results. This variability may reduce the reliability of the findings.

Still, this review provides a useful starting point for further research, which is necessary, particularly in the context of post-pandemic work communities and their resilience in healthcare. The included studies originate from highly diverse cultural and organisational settings and contextual or cultural factors could not be addressed. This may influence the transferability of findings.

## Conclusions

The current evidence indicates that leadership behaviours play a crucial role in preventing and reducing workplace loneliness and social isolation in healthcare. This study identifies specific leadership behaviours, such as seeing the person as a human being, strengthening a sense of belonging, fostering interpersonal relationships and enhancing sustainable working conditions. These behaviours also reflect components commonly associated with established leadership styles, such as transformational, ethical, servant and relational or inclusive leadership. By implementing these behaviours, leaders can promote change within work communities towards a humane, soft, people-centred culture that does not necessarily require additional financial resources. It requires an attitudinal shift towards recognising the value of individual professionals, community, cohesion, reciprocity, participation and sustainability.

### Implications for practice and research

This scoping review comprehensively presents leadership behaviour that can prevent loneliness and social isolation in the healthcare workplace. Loneliness and social isolation affect employees’ well-being, but they are not only individual-level phenomena; they also involve structural and organisational factors that can be influenced through political decision-making. Decision-makers must ensure that leadership responsibilities are clearly defined and that leaders receive education and support to cope with this demanding task. The leader has a vital role in enabling and supporting participation and involvement in the workplace. Leaders’ presence is pivotal for reducing and preventing loneliness and social isolation. Leaders need to support work control, clarify the distribution of work among professionals, provide feedback and develop ways to foster a sense of appreciation and commonality among professionals.

The results indicate that academic research should deepen the theoretical and empirical understanding of workplace loneliness and social isolation. Future studies should investigate the efficacy of tailored interventions to prevent and reduce workplace loneliness and social isolation among healthcare workers and leaders, and examine the contextual variation and long-term effects of leadership on social integration. In addition, future studies should consider the relevance of underexplored concepts such as psychological safety, which might play a significant role in loneliness and social isolation.

## Supplementary Material

Data supplement 1

Data supplement 2

Data supplement 3

Data supplement 4

## Data Availability

The data supporting this study’s findings are available in the supplementary material of this article.

## Reporting method

The Preferred Reporting Items for Systematic Reviews and Meta-analyses extension for Scoping Reviews (PRISMA-ScR) were used for reporting.

## Protocol registration

This protocol has been registered in the Open Science Framework (osf.io/e8yb6).

## Supplementary material

The supplementary material for this article can be found online.
