# Supplementary material for: Leadership behaviour in preventing and reducing workplace loneliness and social isolation in healthcare: a scoping review
Source: Leadersh Health Serv (Bradf Engl). 2026 Apr 2;39(5):33–48. doi: 10.1108/LHS-08-2025-0131 (PMC13052628; doi:10.1108/LHS-08-2025-0131)
Supplement: Data supplement 2 [file lhs-08-2025-0131_suppl2.docx]

Supplementary Table 2. Search strategies and results by databases.

| Database | Search strategy | n |
| --- | --- | --- |
| MedNar  CINAHL | (lead* OR manag* OR supervis* OR administrat*) and (work* OR occupation*) and (loneliness OR isolat* OR "social alienation" OR ostracism) and (health OR healthcare OR nurs* OR hospital* OR medic*)  ((MH "Leadership") OR (MH "Management Styles") OR (MH "Nursing Management+") OR (MH "Nurse Managers+") OR (MH "Nursing Leaders+") OR (MH "Supervisors and Supervision") OR (MH "Administrative Personnel+") OR (MH "Personnel Management+") ) OR ( lead* OR manag* OR supervis* OR administrat* ) ) AND ( (MH "Work+") AND (MH "Social Isolation+")) OR ((MH "Work+") AND (loneliness OR isolat* OR “social alienation” OR ostracism)) OR ( (MH "Social Isolation+") AND (work* OR occupation*)) OR ((work* OR occupation*) N3 (loneliness OR isolat* OR “social alienation” OR ostracism)) ) AND ((MH "Health Personnel+") OR (MH "Health Occupations+") OR (MH "Hospitals+")) OR (health OR healthcare OR nurs* OR hospital* OR medic*)) | 1503  1300 |
| Ovid Medline | (exp Administrative Personnel/ or exp Personnel Management/ or exp Leadership/ or (lead* or manag* or supervis* or administrat*).ab,kf,ti.) and ((exp Work/ and exp Social Isolation/) or (exp Work/ and (loneliness or isolat* or "social alienation" or ostracism).ab,kf,ti.) or (exp Social Isolation/ and (work* or occupation*).ab,kf,ti.) or ((work* or occupation*) adj4 (loneliness or isolat* or "social alienation" or ostracism)).ab,kf,ti.) and (exp Health Personnel/ or exp Hospitals/ or (health or healthcare or nurs* or hospital* or medic*).ab,kf,ti.) | 1340 |
| Scopus | (TITLE-ABS-KEY (lead* OR manag* OR supervis* OR administrat*) AND TITLE-ABS-KEY ((work* OR occupation*) W/3 (loneliness OR isolat* OR "social alienation" OR ostracism)) AND TITLE-ABS-KEY (health OR healthcare OR nurs* OR hospital* OR medic*)) | 1110 |
| ProQuest | noft(lead* OR manag* OR supervis* OR administrat*) AND noft((work* OR occupation*) NEAR/3 (loneliness OR isolat* OR "social alienation" OR ostracism)) AND noft(health OR healthcare OR nurs* OR hospital* OR medic* | 708 |
| Web of Science  Medic | lead* OR manag* OR supervis* OR administrat* (Topic) and (work* OR occupation*) NEAR/3 (loneliness OR isolat* OR "social alienation" OR ostracism) (Topic) and health OR healthcare OR nurs* OR hospital* OR medic* (Topic)  "Administrative Personnel" "Personnel Management" Leadership johta* AND Work työ* ammat* AND "Social Isolation" Loneliness yksinäi* erist* ostrakismi* | 623  16 |

Searches conducted in January 2025, without time limits.

Source: Authors’ own work.
