# Supplementary material for: Leadership behaviour in preventing and reducing workplace loneliness and social isolation in healthcare: a scoping review
Source: Leadersh Health Serv (Bradf Engl). 2026 Apr 2;39(5):33–48. doi: 10.1108/LHS-08-2025-0131 (PMC13052628; doi:10.1108/LHS-08-2025-0131)
Supplement: Data supplement 3 [file lhs-08-2025-0131_suppl3.docx]

Supplementary Table 3. Ineligible studies following full-text review.

| Ineligible studies | Reason for exclusion |
| --- | --- |
| 1. Seeholzer, E., Santiago, M., Thomas, C., DeAngelis, M., Scarl, F., Webb, A., Woods, T. & Sehgal, A. (2022). Prevalence of Social Determinants of Health Among Health System Employees. *Journal of Primary Care & Community Health ,*13. doi: 10.1177/21501319221113956 | Wrong phenomenon  of interest |
| 1. Mahapatro, M. & Prasad, M. (2023). The experiences of nurses during the COVID‐19 crisis in India and the role of the state: A qualitative analysis. *Public Health Nursing,* 40(5). doi: 10.1111/phn.13205. | Wrong phenomenon  of interest |
| 1. Ding, C., Li, L., Li, G., Li, X., Xie, L. & Duan, Z. (2023). Impact of workplace violence against psychological health among nurse staff from Yunnan-Myanmar Chinese border region: propensity score matching analysis. *BMC Nursing,* 22(1). doi: 10.1186/s12912-023-01402-w | Wrong phenomenon  of interest |
| 1. Birt, L., Lane, K., Corner, J., Sanderson, K. & Bunn, D. (2023). Care‐home Nurses' responses to the COVID‐19 pandemic: Managing ethical conundrums at personal cost: A qualitative study. *Journal of Nursing Scholarship,* 55(1). doi: 10.1111/jnu.12855 | Wrong phenomenon  of interest |
| 1. Gilliland, K., Tosch, P., Hussey, L., Hines, C., Lane, L., Loftis, P-A., Orr, M., Perkins, K., Svetlik, D.A. & Mancini, M.E. (1990). Specialty nursing council: a peer support group for nurses in independent roles. *Clinical nurse specialist,* 4 (1), 38-42 | Wrong phenomenon  of interest |
| 1. Flood, T., McFadden, S. & Shepherd, P. (2022). The impact of COVID-19 on the mental health of radiography staff and managers in Northern Ireland, UK: The radiography managers' perspective. *Radiography,* 28(1). doi: 10.1016/j.radi.2022.06.011 | Duplicate |
| 1. Ranasinghe, P. & Zhou, A. (2023). Women physicians and the COVID-19 pandemic: gender-based impacts and potential interventions. *Annals of medicine,* 55(1), 27-34. doi: 10.1080/07853890.2022.2164046 | Wrong study design |
| 1. Krug, H., Haslam, S., Otto, K. & Steffens, N. (2021). Identity Leadership, Social Identity Continuity, and Well-Being at Work During COVID-19. *Frontiers in psychology,* 12. doi: 10.3389/fpsyg.2021.684475 | Wrong participants |
| 1. Zamanzadeh, V., Valizadeh, L., Khajehgoodari, M. & Bagheriyeh, F. (2021). Nurses' experiences during the COVID-19 pandemic in Iran: a qualitative study. *BMC Nursing,* 20(1). doi: 10.1080/07853890.2022.2164046. | Wrong phenomenon  of interest |
| 1. Munir, Y., Khan, S., Sadiq, M., Ali, I., Hamdan, Y. & Munir, E. (2016). Workplace Isolation in Pharmaceutical Companies: Moderating Role of Self-Efficacy. *Social Indicators Research,* 126 (3) 1157-1174. doi: 10.1007/s11205-015-0940-7 | Wrong participants |
| 1. Samushonga, H. (2021). Insights into research based management decision-making in healthcare: Revealing the risk of professional isolation for mobile-working community nurses. *International journal of healthcare management,* 14(3), 906-913. doi: 10.1080/20479700.2020.1719462. | Wrong outcomes |
| 1. Gursoy E, Saglam H, Yavuz N. (2023) Experiences of intensive care nurses working with patients with COVID-19: A qualitative study in Turkey. *Nursing Practice Today* 10(2), 124-137. DOI: 10.18502/npt.v10i2.12833 | Wrong phenomenon  of interest |
| 1. Riley R, Spiers J, Buszewicz M, Taylor A, Thornton G, Chew-Graham C. (2018). What are the sources of stress and distress for general practitioners working in England? A qualitative study. *BMJ open* 8(1). DOI: 10.1136/bmjopen-2017-017361 | Wrong phenomenon  of interest |
| 1. Pang Y, Chen Y. (2022) The Effect of Self-efficacy on Workplace Loneliness under COVID-19: The Chain Mediating Role of Professional Identity and Social Support. *Association for Computing Machinery* 208-215. DOI: 10.1145/3564858.3564892 | Wrong phenomenon  of interest |
| 1. Trueland J. (2022) Building stronger teams: Nurse leadership in social care: The pandemic highlighted the complex role of social care nursing. Now, it is hoped that career pathways and peer support will help to boost its image and attract people to the sector. *Nursing Standard* 37(9), 19-22. DOI: 10.7748/ns.37.9.19.s12 | Wrong study design |
| 1. Weeks L C, Barrett M, Snead C. (1985) Primary nursing. Teamwork is the answer. *The Journal of nursing administration* 15(9), 21-6. | Wrong phenomenon  of interest |
| 1. Cabello M, Izquierdo A, Leal I. (2022) Loneliness and not living alone is what impacted on the healthcare professional's mental health during the COVID‐19 outbreak in Spain. *Health & Social Care in the Community* 30(3), 968-975. DOI: 10.1111/hsc.13260 | Wrong phenomenon  of interest |
| 1. Pei H, Mepham J, Eastwood B, Skinner M A. (2022) An Investigation of the Experiences of Physiotherapists During the Aotearoa New Zealand COVID-19 Pandemic 2020. *New Zealand Journal of Physiotherapy* 50(3):133-149. DOI: 10.15619/nzjp/50.3.05 | Wrong phenomenon  of interest |
| 1. Rolf D. (2016) Life on the Homecare Front. *Journal of the American Society on Aging* 40(1):82-87 | Wrong phenomenon  of interest |
| 1. Fernández‐Castillo R‐J, González‐Caro M‐D, Fernández‐García E, Porcel‐Gálvez A‐M, Garnacho‐Montero J. (2021) Intensive care nurses' experiences during the COVID‐19 pandemic: A qualitative study. *Nursing in Critical Care* 26(5), 397-406. DOI: 10.1111/nicc.12589 | Wrong phenomenon  of interest |
| 1. Merchant J. (2021) How district nurses can support team wellbeing during the pandemic and beyond. British journal of community nursing, 26(7), 318-323. DOI: 10.12968/bjcn.2021.26.7.318 | Wrong study design |
| 1. Pniak B, Leszczak J, Adamczyk M, Rusek W, Matlosz P, Guzik A. (2021) Occupational burnout among active physiotherapists working in clinical hospitals during the COVID-19 pandemic in south-eastern Poland. *Work* 68(2), 285-295. DOI: 10.3233/WOR-203375 | Wrong phenomenon  of interest |
| 1. Connelly D, Garnett A, Snobelen N, Guitar N, Flores‐Sandoval C, Sinha S, Calver J, Pearson D, Smith‐Carrier T. (2022) Resilience amongst Ontario registered practical nurses in long‐term care homes during COVID‐19: A grounded theory study. *Journal of Advanced Nursing* 78(12):4221-4235. DOI: 10.1111/jan.15453 | Wrong phenomenon  of interest |
| 1. Holt, L. (2022). Why don't we talk about loneliness in leadership?: Loneliness can still be seen as a taboo subject in healthcare settings, with those in leadership roles often wanting to appear strong and 'professional'. *Nursing Standard,* 37(12), 46-47. doi: 10.7748/ns.37.12.46.s20 | Wrong study design |
| 1. Pei-Hsuan, Y. & Ying-Hua, T. (2022). Demographic Variables, Perceived Work Stress, and Job Satisfaction as Predictors of Organizational Commitment in Nurses During the COVID-19 Pandemic. *Journal of Nursing,* 69(6), 33-44. doi: 10.6224/JN.202212_69(6).06 | Wrong language |
| 1. Holt, L. (2022). Feeling lonely at the top?: Nurses in leadership roles often want to appear strong and 'professional', and talking about loneliness can still be seen as a taboo. *Primary Health Care,* 32(6), 18-19. doi: 10.7748/phc.32.6.18.s6 | Wrong study design |
| 1. Woods, D., Navarro, A., Borde, P., Dawson, M. & Shipway, S. (2022). Social Isolation and Nursing Leadership in Long-Term Care: Moving Forward After COVID-19. *Nursing Clinics of North America,* 57(2), 273-286. doi: 10.1016/j.cnur.2022.02.009 | Wrong study design |
| 1. Yerushalmi, H. (2020). Play in social workers' psychodynamic therapy supervision. *Journal of Social Work Practice,* 34(3), 297-308. doi: 10.1080/02650533.2019.1692805. | Wrong study design |
| 1. Hills, L. (2016). Loneliness at the Top: Ten Ways Medical Practice Administrators Can Manage the Isolation of Leadership. *Journal of Medical Practice Management,* 31(5), 292-296 | Wrong study design |
| 1. Kanellopoulos, D., Solomonov, N., Ritholtz, S., Wilkins, V., Goldman, R., Schier, M., Oberlin, L., Bueno-Castellano, C., Dargis, M., Cherestal, S. & Gunning, F. (2021). The CopeNYP program: A model for brief treatment of psychological distress among healthcare workers and hospital staff. *General Hospital Psychiatry,* 73, 24-29. doi: 10.1016/j.genhosppsych.2021.09.002 | Wrong phenomenon  of interest |
| 1. Place, A. (2023). How returning to the office can support employee mental health. *Employee Benefit News (Online),* New York SourceMedia 2023. | Wrong study design |
| 1. Johnston, S., Heneghan, P. & Daniels, P. (2020). Mentoring initiative to retain community-based registered nurses in palliative care. *British Journal of Community Nursing,* 25(7), 335-339. doi: 10.12968/bjcn.2020.25.7.335 | Wrong phenomenon  of interest |
| 1. Amarat, M., Akbolat, M., Ünal, Ö. & Güneş, K. (2019). The mediating role of work alienation in the effect of workplace loneliness on nurses' performance. *Bilge Journal of Nursing Management,* 27(3), 553-559. doi: 10.1111/jonm.12710 | Wrong phenomenon  of interest |
| 1. Ali Awad, N.H., Mohamed El Sayed, B.K. (2023). Post COVID-19 workplace ostracism and counterproductive behaviors: Moral leadership. *Nursing Ethics,* 30(7-8), 990-1002. doi:10.1177/09697330231169935 | Wrong study design |
| 1. Manninen, S.M., Koponen, S., Sinervo, T. & Laulainen, S. (2024). Workplace ostracism in healthcare: Association with job satisfaction, stress, and perceived health. *Journal of Advanced Nursing,* 80(5), 1813-1825. doi: 10.1111/jan.15934 | Wrong phenomenon  of interest |
| 1. Klingemann, J., Mokros, L., Sienkiewicz-Jarosz, H. & Świtaj, P. (2024). The prevalence of occupational burnout and its individual and situational predictors among addiction therapists. *Alcohol & Alcoholism,* 59(1), 1-7. doi: 10.1093/alcalc/agad074 | Wrong phenomenon  of interest |
| 1. Riley, K., Wilson, V., Middleton, R. & Molloy, L. (2024). Examining the roles of rural nurses in resuscitation care: An ethnographic study. *International Emergency Nursing,* 73. doi: 10.1016/j.ienj.2023.101404 | Wrong phenomenon  of interest |
| 1. Mohammad, S.S., Mufti, S. & Nazir, N.A. (2024). Management of Psychosocial Risks at Workplaces Amid the COVID-19 Pandemic. *Emerging Business Trends and Management Practices: Lessons from the Pandemic*. 215-232. doi: 10.1201/9781003331353-10 | Wrong phenomenon  of interest |
| 1. Boitet, L., Meese, K., Hays, M., Gorman, C., Sweeney, K. & Rogers, D. (2023). Burnout, Moral Distress, and Compassion Fatigue as Correlates of Posttraumatic Stress Symptoms in Clinical and Nonclinical Healthcare Workers. *Journal of Healthcare Management,* 68(6), 427-451. doi: 10.1097/JHM-D-23-00098 | Wrong phenomenon  of interest |
| 1. Shanafelt, T., Trockel, M., Mayer, T., Wang, H. & Athey, L. (2024). Evaluation of Work-Life Integration, Social Isolation, and the Impact of Work on Personal Relationships Among Healthcare CEOs and Administrative Leaders. *Journal of Healthcare Management,* 69(2), 99-117. doi: 10.1097/JHM-D-23-00134 | Wrong phenomenon  of interest |
| 1. Fang, X-H., Wu, L., Lu, L-S., Kan, X-H., Wang, H., Xiong, Y-J., Ma, D-C. & Wu G-C. (2021). Mental health problems and social supports in the COVID-19 healthcare workers: a Chinese explanatory study. *BMC Psychiatry,* 21(1). doi: 10.1186/s12888-020-02998-y | Wrong phenomenon  of interest |
| 1. Higgins, N., Jones, L., Hutton, T., Dart, N., Fawcett, L. & Muir-Cochrane, E. (2024). Survey of staff experiences of potential stigma during the COVID-19 pandemic. *International Journal of Mental Health Nursing,* 33(4). doi: 10.1111/inm.13284 | Wrong phenomenon  of interest |

Authors’ own work
