# Supplementary material for: Leadership behaviour in preventing and reducing workplace loneliness and social isolation in healthcare: a scoping review
Source: Leadersh Health Serv (Bradf Engl). 2026 Apr 2;39(5):33–48. doi: 10.1108/LHS-08-2025-0131 (PMC13052628; doi:10.1108/LHS-08-2025-0131)
Supplement: Data supplement 4 [file lhs-08-2025-0131_suppl4.docx]

Supplementary Table 4. A summary of the studies included in the review.

| Author(s)  (year)  country | Aim | Study design | Data collection and data analysis | Participants and study settings | Key findings |
| --- | --- | --- | --- | --- | --- |
| Aira et al.  (2010)    Finland | To gain a deeper understanding of feeling isolated in general practices and determine which factors constitute it. | Qualitative study, in-depth interviews | Data collection: One interviewer, who is an expert in qualitative research methods and has extensive experience as a GP in health centers, conducted the interviews, but not in the researched health centers.    The chief physician provided contact information for interviewees, and physicians were purposefully chosen to ensure a diverse sample.    Data Analysis: All data were entered into a QSRNVivo7 computer software package and examined line-by-line by two researchers separately. The main categories and themes were identified and coded using thematic analysis and constant comparison. Saturation was achieved when no new themes emerged. | Primary health care physicians (n = 32, 10 males, 22 females) in six different communities: two small-sized (< 5000 inhabitants), two medium-sized (> 10 000 inhabitants) and two large (> 50 000 inhabitants) communities | Many GPs experienced a sense of loneliness and isolation when they began working in primary health care centers. They faced challenges in transitioning from a hospital environment where they primarily worked in teams to a context where they had to make many decisions independently.    The qualitative data analysis identified four main themes related to isolation: making decisions alone, lack of collaboration with other healthcare workers, not feeling part of the work community, and a lack of mentoring at work.    The study found that these feelings of isolation existed across various levels of health care organization, including relations with the health care system or employer group, specialist health care, colleagues, and the nursing team. GPs often had to make rapid decisions about patient examinations and treatments independently. While they perceived this autonomy positively, there were occasions when they desired consultation with other physicians, especially young doctors seeking guidance. Difficulties in obtaining feedback and adequate communication with hospital specialists were highlighted. Some health centers facilitated consultations through electronic systems or hired private specialists for in-house consultations, which the GPs found beneficial.    Concerns were raised about the distribution of tasks from secondary care to primary care without consideration of the capacity or training needs. Collaboration with nursing staff was also perceived as deficient in some instances. The study identified a perceived distance between primary and secondary health care, with limited shared understanding of working conditions between health center and hospital doctors. |
| Arslan et al. (2020)    Turkey | To examine how social interaction variables  (leader-member exchange (interactions between managers and nurses), trust, and  communication frequency) and work meaningfulness influence nurses' experiences  of workplace loneliness. | Cross-sectional study design | Data collection:  A survey with self-report scales including Leader-Member-Social Exchange Scale, Meaningful Work Scale, Trust in Leader Scale, Loneliness at work Scale, Frequency of Communication Scale    Data analysis: Jamovi 1.0.7.0 was used for the confirmatory factor analysis of the translated scales. Results are presented in descriptive statistics, interscale correlations and reliability estimates (Cronbach’s alpha). | Turkish nurses (n = 874) | A negative correlation between loneliness and the Leader-Member Social Exchange (LMSX), Trust in the Leader, Meaningful Work and Frequency of Communication could be found. Both trust in the leader and work meaningfulness significantly negatively influence workplace loneliness. A higher frequency of communication reduces workplace loneliness, according to the moderation model. Trust in a leader and work meaningfulness are two partially mediating variables in workplace loneliness and leader-member social exchange. Trust in leader and work meaningfulness significantly and negatively predicted workplace loneliness. The total effect of LMSX on workplace loneliness was significant. The indirect effect of LMSX on loneliness, via both trust in the leader and meaningfulness of work, was significant.    Both low and high communication frequency strengthened the positive relationship between trust in leader and workplace loneliness. When communication frequency is higher, perceived workplace loneliness decreases more. Both trust in the leader and work meaningfulness partially mediated the effect on LMSX on workplace loneliness.    The following hypotheses were supported:  Employees who interact more with their leader will  have greater trust in their leader.  Trust in the leader reduces the sense of loneliness.  Trust in the leader has a mediating effect on loneliness.  - increase the effect of social interaction and trust  in the leader (moderation)  - increase the effect of social interaction and the  perception of meaningful work (moderation  - increase the effect of meaningful work on  loneliness (moderation)  Work meaningfulness will have a negative  relationship with loneliness  Work meaningfulness will mediate the effect  between social exchange and loneliness  Loneliness is higher for nurses with low interaction with their leaders, low trust in leaders and low work meaningfulness. -> “Through creating trust, communicating frequently and providing a meaningful work environment, managers can reduce the loneliness experienced by nurses.” |
| Cho & Kim (2024)    Korea | To understand the experiences of nurses working in the integrated nursing care service to suggest ways in which to address their grievances and needs and improve their job satisfaction, thereby reducing turnover. | A phenomenological study | Data collection:  In-depth interviews with nurses working in integrated nursing care wards.    Data analysis:  Colaizzi’s phenomenological research method | A total of 17 nurses having more than one year of working experience in an integrated service ward.    All the participants were women, and their mean age was 31.0 ± 5.2 years. Their mean total clinical work experience was 7.19 ± 5.32 years, and their mean experience in the integrated nursing care ward was 1.97 ± 0.43 years. | One of the theme clusters identified was loneliness and fighting alone. Nurses working in the integrated nursing service ward did not feel that they were cooperating with people in different occupations. Even when a patient suffered a fall, all the responsibility was placed on the nurse. The doctors who visited the ward also wanted the nurses to fulfill all the needs of the patients, assuming that that was the role of a ward nurse. |
| Crawford et al. (2005)    United States | To “characterize the work environment and identify factors that influence the occupational health of dental hygienists.” | Qualitative  Research, interview for semi-structured focus groups | Data collection: focus groups conducted in June 2003.      Data analysis: Analysis was conducted using  standard methodology. The unit of analysis was the individuals. Data coding was done using a combination of note-based and tape-based methodologies. | Dental hygienists (n = 51) | Social isolation was mentioned, as dental hygienists seem to feel less likely to be integrated into an office than other dental workers, mainly because they have several part-time jobs and are not employed in one office. Thus, the feeling of being “treated like contract workers” was mentioned, as well as a lack of professional peers. The lack of breaks enhances the feeling of social isolation.    Differences in work structure and pay create a chasm between hygienists and other staff. Other office staff do not understand their responsibilities or education level and, therefore, do not understand why the hygienists earn much more. The other office staff may show their resentment by refusing to assist the hygienists in any of their work or by scheduling them heavily.    Although hygienists see patients alone, dental assistants work directly with dentists. The hygienists felt that dentists often seem to favour the assistant in times of office conflict, which adds to social isolation.    Relationships between dentists and assistants impact solving conflicts in workplaces, and hygienists often feel in an unequal position compared to family members or spouses in work communities. |
| Digby et al. (2021)    Australia | To identify the effect and challenges of working during Covid-19 on the wellbeing of hospital staff and inform about approaches to improve the working conditions. | An exploratory, qualitative study as part of a larger mixed-method research project | Data collection: online survey.    Data analysis: inductive content analysis for the free-text questions. | Clinical staff, including medical, nursing and allied health and non-clinical staff, working at one acute hospital during the COVID-19 pandemic (n=321). Most participants were clinical staff (senior  medical staff (SMO) (n = 58), junior medical staff (JMO) (n = 41), nurses (n = 86), and allied health staff (n = 103) and some non-clinical participants (n = 33). | During the pandemic, many staff were instructed to work from home and use telehealth, teleconferences, email, or phone to work remotely to maintain social distancing. Regarding social isolation and social distancing, some clinicians, especially those living alone, experienced loneliness, reduced motivation and dissatisfaction. Due to government restrictions and social isolation, positive mental health resources such as friends, family or leisure time were not accessible anymore and thus added to the stressful work during the pandemic for hospital staff, leading to even more social and emotional isolation.    Nurses working in COVID-19-positive isolation rooms were required to wear full protective outfits for the whole shift. They reported that it was often unpleasant, restrictive, and isolating.    Managers played an important role in directing correct processes, listening to staff, and addressing issues during COVID-19 pandemic when staff perceived social and emotional isolation and social distancing. |
| Dreachslin et al. (2000)    United States | To examine the detailed effects of racial diversity on self-perceived effectiveness of communication in nursing care teams | Qualitative study design:  14 focus groups | Data collection: 14 focus groups with audiotaped verbatim transcripts    Data analysis: Transcript-based analysis to develop a grounded theory | Nursing care teams of two hospitals in the northeastern USA.  The number of participants was not reported. | With more racial diversity, more different perspectives and alternative realities arise in a team. The grounded theory analysis identified three reinforcing factors that increase miscommunication: social isolation, selective perception, and stereotypes. Social isolation may occur due to the (in)voluntary clustering of ethical groups within a team.    Leaders who reduce social isolation by creating opportunities for interracial social interaction and who, through example, demonstrate self-monitoring behavior that mitigates selective perception and stereotyping increase communication effectiveness in nursing care teams. Diversity leadership thus serves as the mitigating factor in the relationship between race and the effectiveness of self-perceived communication in nursing care teams. |
| Flood et al.  (2022)    United Kingdom | To capture perceptions of diagnostic and therapeutic radiography managers regarding the impact of COVID-19 on their and that of their staff mental health | Qualitative study  (mixed methods, qualitative results reported) | Data collection: Phase 1 involved an electronic survey, Phase 2 involved qualitative interviews  In Phase 2, qualitative data was collected through semi-structured online interviews with ten radiography service managers from Health and Social Care trusts and the private sector.    Data analysis: Qualitative data was analyzed using NVivo software. Data was independently coded by 2 researchers, using an  iterative approach following Braun & Clarke’s six-stage framework for thematic analysis. | In total, 11 interviews were conducted. Eight Diagnostic radiography (DR) service managers from across the five Trusts and private sector participated in interviews, some of whom also oversaw services other than diagnostic radiography. Two Therapeutic Radiography (TR) service managers representing the radiotherapy departments participated in the interviews. Additionally, discussions between managers and their team leaders resulted in a superintendent therapeutic radiographer being interviewed. | The study highlights the negative factors affecting mental health during the pandemic, such as changing PPE guidelines, social distancing measures, lunchtime separations, and service restructuring. These factors increased anxiety and fear among staff and managers, leading to confusion and fatigue. However, positive factors such as staff resilience, strong work ethics, and support among multidisciplinary teams positively influenced mental health. Managers also appreciated the increased focus on family life and work-life balance during the pandemic. They supported mental health by accommodating staff needs, promoting communication, and implementing regular ‘huddles’ to address concerns. Open-door policies and team events also aimed to boost morale. However, trust support services were recognized but healthcare professionals prioritized patient care over seeking support for themselves. |
| Førsund & Schumacher (2023)    Norway | To explore and describe how nurses experienced working in home care during the COVID-19 pandemic | Qualitative descriptive–interpretative design | Data collection:  A semi-structured individual interview  Data analysis:  Systematic text condensation inspired by Malterud. Inductive method for performing thematic cross-case analysis on qualitative data. | Nine (9) interviews were conducted for home care nurses, who worked in municipal home care with varying levels of work experience and age from March 2020 and throughout the pandemic from different municipalities in Southeast Norway | Two categories describing nurses´ experiences during COVID-19 were identified through analysis: (1) adapting approaches and (2) adapting work practices.    Adapting approaches included navigating isolation and responsibility based on nurses' sense of isolation when facing challenging decisions in patient situations and organizational inquiries while providing care at the patient's homes. Nurses felt they lacked support from the leaders working remotely and from the medical facilities, such as hospitals and doctors.    Adapting approaches included ensuring personal and collective safety, which means that nurses isolated themselves socially as they imposed restrictions on their personal lives. |
| Huyghebaert et al.  (2018)    France | To investigate the relationship between perceived career opportunities, affective commitment to the supervisor, and social isolation from colleagues on the one hand and nurses’ well-being and turnover intentions on the other  To explore the mediating role of affective commitment to the organization in these relationships | Quantitative study, cross-sectional study design | Data collection:  a questionnaire, nurses were surveyed with the approval of their organization    ACS was measured with Vandenberghe and Bentein’s (2009) six-item scale  Social isolation was assessed with the six-item colleagues' subscale (a=0.92) from the workplace isolation scale developed by Marshall et al. (2007)  PCO were measured with three items (a=0.85) developed by Kraimer et al. (2011)  ACO was assessed with the six items (a=0.86) used by Bentein, Vandenberg, Vandenberghe, and Stinglhamber (2005)  Well-being was measured through the five-item high pleasurable-high arousal subscale from the job-related affective well-being scale (Van Katwyk, Fox, Spector, & Kelloway, 2000)    Data analysis:  Confirmatory factor analysis using a covariance matrix  Hypotheses were tested using path analysis procedures  Bootstrapping approach | 244 nurses from various French health care centres (e.g. public hospitals, nursing homes), between 20 and 59 years. The average tenure in the current health organization was 3.91 years (SD=5.27), and the average tenure in the current job was 2.56 years (SD=3.67). 220 (90.2%) of them were full-time workers, 182 (74.6%) permanent nurses, 62 (25.4%) temporary workers, 16 (6.6%) worked in organizations with 11-49 employees, 60 in organizations with 50-249 employees (24.6%), 29 in organizations with 250-499 employees (11.9%) and 139 in organizations with over 500 employees (57.0%) | The results show that affective commitment to the supervisor (ACS) and perceived career opportunities (PCO) are positively related to commitment to the organization (ACO), well-being and turnover intentions, while workplace isolation is negatively linked to ACO and well-being but positively associated with turnover intentions. ACO is positively linked to well-being and negatively associated with turnover intentions. The study also found that social isolation from colleagues negatively affects well-being and increases turnover intentions among nurses. Stronger ACO correlates with reduced turnover intentions and higher well-being. ACO partially mediates the relationship between ACS, PCO, social isolation, and both well-being and turnover intentions. The study highlights the role of ACO as a key factor mediating these effects. |
| Lindgren et al. (2001)    Sweden | To explore how physiotherapist, working within primary healthcare in the city of Umeå, Sweden, perceive their psychosocial work environment | Qualitative interview study | Data collection: The data were collected in 2000 through face-to-face thematic interviews. The respondents were selected by snowball sampling from healthcare centers in the Umeå region.    Data analysis: The interviews were analyzed by the grounded theory method | Physiotherapists (n = 4) | Physiotherapists described themselves as outsiders in the primary healthcare organization, regarding their actual role in primary healthcare and in relation to other healthcare professionals. Physiotherapists worked in physically different buildings or on different floors from where the actual work was done, which caused loneliness. Often the physiotherapists' managers had a different professional background, which meant they did not feel able to ask their manager for help with work-related issues. Physiotherapists perceived themselves as outsiders in primary healthcare organizations due to their different professional backgrounds. Physiotherapists work in collaboration with a multidisciplinary team. Multidisciplinary collaboration works in different ways depending on the personalities of the professionals in the team and how well their personalities fit together. In addition, feelings of loneliness at work were related to the isolated nature of the job. In their own work, they found themselves alone with the patient’s problem, without anyone to seek advice from. However, physiotherapists perceived that they received support from other physiotherapists, but only if the cooperation worked well. Physiotherapists expressed their fear of demonstrating their own competence deficits. Most of them hoped for ongoing professional guidance to help them overcome difficulties in their work and improve their professional development. |
| Mäntyselkä et al.  (2010)    Finland | To gain a deeper understanding of the factors related to the perceived isolation of physicians in a health centre in Finland. | Quantitative study design, | Data collection: postal questionnaire    Data analysis: differences  between the groups were assessed using the chi-square test | physicians who worked in a health centre in 2000 were included (N = 1829)    57% (N = 1049) were females, mean age was 46 years (range 27–64 years) | A total of 1215 physicians agreed to the main question: working as a doctor in a health centre is too often isolated work. Loneliness is reported more often in females. Less experienced doctors perceive more isolation than more experienced physicians. Isolation was perceived most in bigger towns and less often in municipalities (populations ranging from 5000 to 20,000). Physicians in large health centres reported the feeling of isolation more often than physicians in small health centres. Poor collaboration with colleagues or other collaborators and infrequent opportunities to consult with colleagues were associated with perceived isolation at work. |
| Stoica. et al (2014)    Romania | To identify the relationship between variables of recognition and attachment to the organization and supervisory support and loneliness at the workplace in healthcare | Quantitative survey study design | Data collection: Loneliness at work scale, structured questionnaire to identify how much the employers perceive the support offered by the superiors at work,  demographic questionnaire,  Pressure Management Indicator    The Loneliness at Work Scale Alphas = 0.92, Mean = 53.5, SD = 19.09.  This is an interview structured around 3 questions to identify how much the employers perceive the support offered by their superiors at work.  A demographic questionnaire: age, gender, level occupied in the organizational hierarchy and seniority in the organization.  The Pressure Management Indicator includes items for attachment to the organization and recognition.  Data analysis: Statistical data analysis. Results are presented as indicators of central tendency for the variables, Pearson correlation between variables, Linear regression analysis of predictor-variables, the multilinear simultaneous regression analysis, Levene, ANOVA and Hochberg’s GT2 test | 138 employees of a medical unit in Romania: 80.4% women, 19.6% male | A significant positive correlation between recognition of achievements and loneliness at the workplace (r = 0.360, df = 136, p < 0.01) and a significant negative correlation between the attachment to the organization and workplace loneliness (r = - 0.402, df = 136, p < 0.01). In this study, those who showed a high level of loneliness also showed a low level of attachment to the organization. Recognition of the work showed a significant statistical and practical link to 12% of the staff to reduce workplace loneliness. In a linear regression model, recognition is a significant predictor of workplace loneliness (F = 20.23, p < 0.01) and attachment is a strong negative predictor  for loneliness (F = 26.18, p < 0.01).  LW is greater for employees who feel a lack of support provided by superiors (Hochberg’s GT2 = -20.51, p = 0.01) compared with the loneliness scores for employees who are satisfied (Hochberg’s GT2 = 18.70, p = 0.01) or neutral (Hochberg’s GT2 = 20.51, p = 0.01) to the support offered by seniors. The relationship between the lack of support provided by senior and LW coefficient of determination r2 = 0.21 considers that 21% of the variance of the two variables have a common trend. |
| Wood et al. (2023)    United States | To investigate the phenomenon of nurse loneliness and the potential contribution of nurse loneliness to  burnout. | Sequential explanatory mixed-methods design: (1) cross-sectional descriptive approach, (2) qualitative semi-structured interview design | Data collection: four geographically diverse hospitals    (1) Quantitative data collection: via online tool REDCap; included demographic data, the Oldenburg Burnout Inventory, revised University of California Los Angeles Loneliness Scale for loneliness assessment  (2) Qualitative data: semi-structured interviews framed in a social constructivist worldview. Interpretive description inquiry was used to gain a richer understanding of nurse loneliness    Data analysis:  (1) Descriptive statistics for summarizing demographic, loneliness, and burnout data. Data were analyzed using JMP software.    ANOVA and chi-square for testing intra-site differences; Pearson’s coefficient for estimating the strength of the relationship, simple linear regression for assessing the relationship between loneliness and burnout; multiple regression model for potential covariates    (2) Interviews: Interpretive descriptive analysis for transcripts analysis independent, team-based and peer debriefing techniques were applied for dependability | 128 registered acute care nurses providing direct patient care at least 32 hours per  two-week period with at least one year of nursing experience    (1) 117 participants,  (2) 11 participants  Participants were predominantly white (80  %), female (85 %), working day shift (66 %), and not participating in a  mentorship program (74 %) | (1): 61 % of participants reported burnout scores ≥ 44, and 34 % reported loneliness scores ≥ 22. A moderate positive correlation between loneliness and burnout (r = 0.55, p < 0.0001) could be found. Loneliness was found to be a predictor of burnout.    (2): Five themes were revealed from the interviews: the destabilization of direct-care nursing that the COVID pandemic has highlighted; the experience of loneliness as feeling unseen and not understood; the emotions of burnout including detachment and a mismatch between ideals and reality; a feeling of dehumanization; and a sense of hope and empowerment in peer connection    The experience of loneliness as feeling unseen and not overall physical and emotional exhaustion was reported.    Many nurses feel unseen as human beings by administrators and leaders in hospital units. Many participants reported feeling lonely both in work and social situations, describing this primarily as feeling unseen and not feeling understood. Several participants referred to nursing shifts as isolating from typical social conventions.    Loneliness appears to be present in nurses and related to burnout. |
| Yeh  (2019)    United Kingdom | To report findings related specifically  to challenges and their impact experienced by homecare workers,  as well as the sources of support used and needed. The paper is part of a wider study which aims to explore homecare workers' views and experiences of providing care to people with dementia up to the end of life. | Qualitative study design | Data collection: face-to-face semi-structured interviews    Data analysis: Transcription of interviews and the framework method of analysis were applied | 29 homecare workers and 13 homecare managers working at a homecare agency in Greater London and South England (N = 42)    More female than male participants, aged between 25 and 50 years; representative to the social care workforce in England | Four main challenges were identified: working with clients with dementia, caring for the dying, conflict with family members, and working alone. Support strategies are preparedness for death, professional support provided by the employer, and peer support.    Homecare is, in general, an isolated setting. Many participants reported that sole working put additional  strains on already pressurized situations, sometimes leading to overwhelming exhaustion, fatigue, and a sense of isolation.    Some homecare managers reported cultivating “open door” practices where staff could “drop in” or call anytime, but overall approaches to supervision and staff support varied across agencies. There also seems to be a discrepancy between what managers said was available and what homecare workers perceived as accessible. Generally, little emotional support appeared to be available. Only in one agency was counselling available, although homecare workers generally had to ask for it or seek it out themselves. Most of the employees would like to have more group meetings or opportunities to interact.  Some managers acknowledged the emotional toll that working with clients with dementia at the end of life had on their staff and described providing avenues for reflective supervision on an ad‐hoc basis or, much less often, experts to help staff manage their distress.  Without professional support, informal networks amongst homecare workers have evolved as a way of connecting and supporting each other. Forming supportive bonds with other homecare workers also helped to alleviate some of the loneliness experienced on the job, as well as facilitated knowledge sharing  between new and more experienced staff. |
| Yousef et. al. (2024)    Saudi-Arabia | Understand the factors that affect the  wellbeing of healthcare professionals in the Kingdom of Saudi Arabia using Job-Demand  and Resource (JD-R) Model | Quantitative study. A cross-sectional, descriptive study. | Data collection: Online survey in English using Google Forms    Data analysis: Descriptive statistics, SPSS | 276  healthcare workers from hospitals and primary healthcare centers, including healthcare professionals, health associate professionals, personal care workers, health management and  support personnel, health service providers, and other healthcare workers | Loneliness was also found to positively impact burnout (β = 0.27, t = 5.547, p <0.01) with an effect size of 0.113 (H2). Workload has a large effect size in comparison to loneliness, having a medium effect size. The hypothesis of a negative relationship between loneliness and work engagement (H4) (β = -0.17, t = 2.096, p < 0.05) with an effect size of 0.017 was supported. |
| Zomerdijk et al., (2023)    Australia | To describe the experiences and needs of healthcare  providers caring for hematology patients during and beyond the COVID-19 pandemic. | Qualitative interview study. | Data collection: A semi-structured interview via telephone.    Data analysis: Data were analyzed using Braun and Clarke's reflexive thematic analysis method. | 21 hematology healthcare  providers (nurses 38.2%, social workers 33.3%, hematologists 14.3%, psychologists 9.5% and exercise physiologists 4.8%) | HCPs highlighted the negative consequences of physical distancing requirements, which impacted on the essential social aspects of their work lives.    “It felt pretty isolating for a long time, and I think that would probably be the biggest impact on my mental health was just that working in a silo” |

 Authors’ own work
